# Complex Vascular Reconstruction for Laparoscopic Right Side Donor Nephrectomy

**DOI:** 10.1155/crit/5387595

**Published:** 2025-05-29

**Authors:** Anupama Murthy, Laura Nino-Torres, Raga Dilip, Marie Le, Jay A. Graham

**Affiliations:** ^1^Department of Surgery, Montefiore Medical Center, Bronx, New York, USA; ^2^Albert Einstein College of Medicine, Bronx, New York, USA

## Abstract

Kidney transplant is the gold standard for the treatment of end-stage renal disease (ESRD). However, there is a significant discrepancy between donor availability and the number of potential recipients on the waiting list. Living donor kidney transplantation has been considered an alternative to increase the donor pool. Left donor nephrectomy is typically preferred due to the length of the renal vein. However, in some cases, right donor nephrectomy must be considered, which presents challenges due to the shorter renal vein and, in some cases, multiple renal arteries. For these cases, transplant surgeons must have alternative strategies to reconstruct the vasculature and ensure that graft implantation and anastomosis are as safe as possible. We present a case of a living donor right laparoscopic nephrectomy with two renal arteries, including vein elongation with an end-to-end anastomosis with a deceased donor renal vein and an end-to-side arterial anastomosis using a deceased donor iliac artery conduit.

## 1. Introduction

There are approximately 90,000 patients on the waiting list for renal transplantation in the United States. annually. However, only 17,000 patients receive an organ, while 100,000 people remain on the waiting list. About 1 in 5 deceased donor kidneys are discarded during the procurement process, exacerbating the demand for organs. Living donor renal transplantation can help reduce wait times, and minimally invasive techniques have made it more appealing to potential donors. However, a patient's access to a living donor is variable. Utilizing living donors with kidneys that have anatomic anomalies may further expand the donor pool. In this report, we describe a donor with two right renal arteries, including a separate lower pole artery, and two right renal veins, which required back table vascular reconstruction prior to transplantation.

## 2. Case Presentation

**Figure 1:**
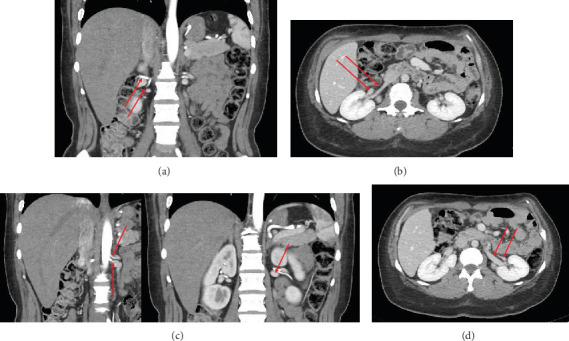
(a) CTA—arteries of right kidney. (b) CTA—veins of right kidney. (c) CTA—arteries of left kidney. (d) CTA—veins of left kidney.

The patient was a 65-year-old female with chronic kidney disease Stage 4, secondary to diabetes, and a prior history of a remote right nephrectomy. She was evaluated to undergo a preemptive living donor kidney transplant from her daughter. The donor was a 37-year-old female with a history of laparoscopic Roux-en-Y gastric bypass surgery and abdominoplasty. Preoperative computed tomography angiography (CTA) revealed two right renal arteries arising from a common branch (Figure 1a), including a separate lower pole artery, and two right renal veins entering separately into the inferior vena cava (IVC) (Figure 1b). The left kidney had three arteries arising from two main renal arteries off the aorta (Figure 1c) and two veins converging in the abdomen prior to entry into the IVC (Figure 1d).

The left kidney was deferred for nephrectomy as the multiple arteries implied a small diameter and increased risk of thrombosis. On the other hand, elongating the right renal vein carries less of a risk for the recipient and the graft. In cases in which we use a right kidney with one artery and one vein, we still use a venous interposition graft to make the venous anastomosis safer and tension-free.

## 3. Surgical Procedure

**Figure 2:**
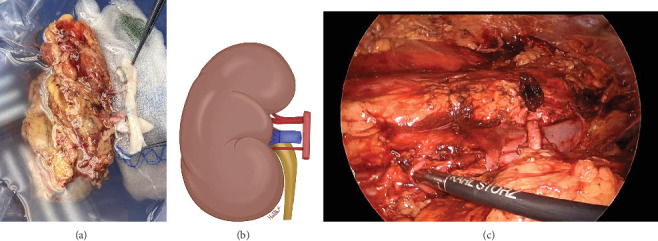
Right kidney after laparoscopic nephrectomy, vascular reconstructions. (a) Surgical photograph of the back table vascular reconstructions of the artery and the vein. (b) Schematic of the end-to-end vein reconstruction with deceased donor vein and end-to-side anastomosis of main renal artery and inferior polar artery to deceased arterial iliac conduit. (c) Medialized living donor kidney with alternating inferior renal artery, inferior renal vein, superior renal artery, and superior renal vein.

A transperitoneal total laparoscopic right donor nephrectomy was chosen and performed after written informed consent. After mobilization of the right kidney, a laparoscopic vascular stapler was used to transect both arteries and both veins as medially as possible to ensure adequate length (Figure 2c).

Upon examination on the back table, the main renal artery, measuring 3.5 mm in diameter, was identified, along with a 1-mm diameter lower pole artery. Arterial reconstruction was performed via end-to-side anastomosis between the 3.5-mm main artery and a donor iliac artery using interrupted 7-0 Prolene sutures and between the 1-mm lower pole artery and the same donor iliac artery using interrupted 8-0 Prolene sutures. The lower pole artery was preserved to ensure perfusion to the ureter. The lower pole renal vein was stapled off. The main renal vein was 4 cm in length and was anastomosed to the deceased donor iliac vein in an end-to-side fashion using 6-0 Prolene sutures to minimize tension along the anastomosis to the recipient (Figure 2a,b). Warm ischemia time was 34 min. Cold ischemia time was 166 min, largely due to the backtable reconstruction.

The deceased donor iliac vessels are obtained through prior pancreas and liver procurements; their availability is a product of collaboration between multiple transplant centers within our region. Donor iliac grafts are verified for ABO compatibility to ensure that they do not increase the risk of rejection. UNOS is also verified for serologies of the specific donor. No virtual crossmatch was done.

## 4. Recipient Postoperative Course

The recipient had an uneventful operative course with Simulect and methylprednisolone induction per institutional protocol. Posttransplantation renal duplex showed patent renal vasculature with adequate velocities and flows. Resistive indices were in normal ranges. Normal venous flow was demonstrated without thrombus. She was started on an infusion of unfractionated heparin postoperatively and transitioned to oral apixaban 2.5 twice daily for 3 months per institutional protocol. She was discharged on postoperative Day 3, at which time her creatinine was 0.78 mg/dL and eGFR was 84 mL/min/BSA from admission Cr of 5.15 and eGFR of 9. She was making 2 L of urine daily. At her 5-month follow-up, her Cr is 0.85, and her eGFR is 76. A repeat renal duplex at 4 months showed continued patency of both arteries without stenosis and patent vein without thrombus. Per institutional protocol, patients with extensive vascular reconstruction are followed with a postoperative duplex and a 3-month course of Eliquis. There is a low threshold to repeat imaging if there is a laboratory or clinical concern.

## 5. Discussion

Traditionally, the left kidney is preferred for living donor renal transplantation due to the increased length of the left renal vein, which allows for easier reimplantation to the recipient's vessels [1]. The choice of kidney laterality is based on optimizing the donor's native kidney function and minimizing side effects, while ensuring recipient graft function [1]. If the left kidney has vascular anomalies, the right kidney should be considered for donation. Right nephrectomy is considered more challenging due to the complexity of achieving adequate length of the right renal vein [2], which is crucial for preventing thrombosis [3] and delayed graft function [2]. However, several techniques allow transplant surgeons to preserve sufficient length of the renal vein, resulting in similar outcomes to left allografts [1].

Protocol renal imaging is a key component of living donor evaluation, as it not only defines the surgical anatomy but also detects occult pathology [4]. In a study assessing renal vascular anatomy on multidetector computed tomography in living donors, conventional vasculature was found in 70.6% of left kidneys and 69.4% of right kidneys [3]. More importantly, only 54.4% had single arteries bilaterally [5]. Although multiple renal arteries (MRA) are found in 18%–43% of potential living donors [4], kidneys with MRA have historically been excluded from living donation [6]. The use of allografts with MRA has increased in the era of laparoscopic donor nephrectomy [5], as the ability to use these grafts improves the donor pool and helps address transplant waiting times [7]. With appropriate laparoscopic surgical techniques and preservation of graft vascular anatomy through ex vivo reconstructions, right donor nephrectomies and MRA allografts should be considered [2]. Therefore, knowledge of arterial reconstruction techniques is fundamental for kidney transplantation [8].

Techniques for revascularizing MRAs include side-to-side or end-to-side anastomoses of two arteries, depending on the caliber of the arteries [9], double distinct anastomoses on external and/or internal recipient iliac arteries, and ligation of small upper pole arteries [2, 10, 11] or ligation of the recipient's internal iliac artery [10]. Renal vessel extension with cryopreserved vascular grafts is also an option, providing vessel extension and faster implantation time [11].

Follow-up of kidney transplant recipients with MRA allografts has been associated with lower 1-year graft survival, higher complication rates, and increased delayed graft function [9, 10]. However, Zorgdrager et al. showed that MRA allografts had no effect on 5-year graft survival or 1- and 5-year patient survival when considering both open and laparoscopic procurements [12]. Some studies have shown that right living donor allografts have more postoperative complications [1], but only 2.2% of MRA graft arterial complications were reported [1], and 4.4% of ureteral complications were reported (vs. 2.6% in single renal artery allografts), which could be related to injuries to inferior polar arteries supplying the ureter [2].

In a meta-analysis of 24 studies evaluating laparoscopic procurement of MRAs versus single-artery kidneys, Afriansyah et al. found no significant difference in donor operative times, length of stay, or complications, similar to other studies [13], suggesting that donor laparoscopic nephrectomy in MRA cases is a safe option [9]. In the recipient group, there were significantly longer cold ischemic times for MRAs requiring back table reconstruction. However, there was no significant difference in delayed graft function, indicating that the longer cold ischemic time did not significantly affect graft function [9]. The recipient group experienced more vascular and ureteral complications and higher one-year graft loss in the MRA group, which differs from the study by Zorgdrager et al. [12]. In a similar meta-analysis, Lim et al. included individual patient data from several studies comparing MRA and single-renal artery (SRA) grafts, concluding that there was no significant difference in long-term graft function or recipient survival between the two groups [14].

The clinical impact of MRAs on recipient outcomes has not been clearly delineated in the literature. However, it is reasonable to conclude that an MRA kidney should not be automatically discarded or excluded from the donor pool. The decision to implant an allograft with MRA should be an individualized choice based on the patient and donor.

## 6. Conclusion

As the gap between available organs and waitlisted patients continues to widen, strategies to increase the donor pool are of paramount importance. The need for a right donor nephrectomy was once considered a contraindication for donation, but it is now viewed as a viable option. Vascular reconstruction in such cases ensures the procedure is safe for the recipient, with no negative impact on patient or graft survival.

## Data Availability

Data sharing is not applicable to this article as no new data were created or analyzed in this study.
